# First Report of OXA-181-Producing *Enterobacterales* Isolates in Latin America

**DOI:** 10.1128/spectrum.04584-22

**Published:** 2023-04-06

**Authors:** Diego Cuicapuza, Luis Alvarado, Norah Tocasca, Daniel Aguilar, Juan Carlos Gómez-de-la-Torre, Guillermo Salvatierra, Pablo Tsukayama, Jesús Tamariz

**Affiliations:** a Facultad de Medicina, Universidad Peruana Cayetano Heredia, Lima, Peru; b Laboratorio de Resistencia Antibiótica e Inmunopatología, Universidad Peruana Cayetano Heredia, Lima, Peru; c Laboratorio de Genómica Microbiana, Facultad de Ciencias y Filosofía, Universidad Peruana Cayetano Heredia, Lima, Peru; d Emerge (Emerging Diseases and Climate Change Research Unit), Facultad de Salud Pública y Administración, Universidad Peruana Cayetano Heredia, Lima, Peru; e Laboratorio Clínico Roe, Lima, Peru; f Instituto Nacional de Enfermedades Neoplásicas, Lima, Peru; g Instituto de Medicina Tropical Alexander von Humboldt, Universidad Peruana Cayetano Heredia, Lima, Peru; h Parasites and Microbes Program, Wellcome Sanger Institute, Hinxton, United Kingdom; JMI Laboratories

**Keywords:** *bla*_OXA-181_, carbapenemase-producing *Enterobacterales*, IncX3 plasmid, Peru, carbapenemase-producing *Enterobacteriaceae*

## Abstract

We characterized five carbapenemase-producing *Enterobacterales* (CPE) isolates from two health care institutions in Lima, Peru. The isolates were identified as Klebsiella pneumoniae (*n* = 3), Citrobacter portucalensis (*n* = 1), and Escherichia coli (*n* = 1). All were identified as *bla*_OXA-48_-like gene carriers using conventional PCR. Whole-genome sequencing found the presence of the *bla*_OXA-181_ gene as the only carbapenemase gene in all isolates. Genes associated with resistance to aminoglycosides, quinolones, amphenicols, fosfomycins, macrolides, tetracyclines, sulfonamides, and trimethoprim were also found. The plasmid incompatibility group IncX3 was identified in all genomes in a truncated Tn*6361* transposon flanked by ΔIS*26* insertion sequences. The *qnrS1* gene was also found downstream of *bla*_OXA-181_, conferring fluoroquinolone resistance to all isolates. CPE isolates harboring *bla*_OXA_-like genes are an increasing public health problem in health care settings worldwide. The IncX3 plasmid is involved in the worldwide dissemination of *bla*_OXA-181_, and its presence in these CPE isolates suggests the wide dissemination of *bla*_OXA-181_ in Peru.

**IMPORTANCE** Reports of carbapenemase-producing *Enterobacterales* (CPE) isolates are increasing worldwide. Accurate detection of the β-lactamase OXA-181 (a variant of OXA-48) is important to initiate therapy and preventive measures in the clinic. OXA-181 has been described in CPE isolates in many countries, often associated with nosocomial outbreaks. However, the circulation of this carbapenemase has yet to be reported in Peru. Here, we report the detection of five multidrug-resistant CPE clinical isolates harboring *bla*_OXA-181_ in the IncX3-type plasmid, a potential driver of dissemination in Peru.

## OBSERVATION

Members of the family *Enterobacterales* are commensals in the intestinal tract and are considered opportunistic pathogens of humans and animals (1). More than 60% of all antibiotics used to treat enterobacterial infections are β-lactams (2). Several resistance mechanisms to β-lactams have been reported, with β-lactamase production being the most common (3). Carbapenems are β-lactams that are highly resistant to degradation by β-lactamases (4). Various carbapenemase-producing *Enterobacterales* (CPE) isolates have been identified, and their spread represents a public health risk worldwide (5).

The KPC, NDM, IMP, VIM, and OXA enzymes are the most common carbapenemases worldwide (6). OXA β-lactamases (class D β-lactamases or oxacillinases) are divided into four groups; OXA-48-like enzymes are included in group III, can hydrolyze carbapenems, and are poorly inhibited by clavulanic acid, tazobactam, and sulbactam (7–9). They show high-level hydrolytic activity against penicillins and low-level hydrolysis of carbapenems with a strong preference for imipenem (10). Previous studies have reported OXA-48-like carbapenemase-producing *Enterobacterales* isolates in humans, animals, food products, and environmental sources (11). Forty-eight OXA-48-like variants have been described (https://www.ncbi.nlm.nih.gov/pathogens/refgene/#oxa-48), with OXA-181 being the second most common (12). It contains four substitutions (the Thr-to-Ala change at position 104 [Thr104Ala], Asn110Asp, Glu168Gln, Ser171Ala) compared to *bla*_OXA-48_ and was first identified in a Shewanella xiamenensis isolate in India (13).

From June 2019 to September 2021, five carbapenem-resistant enterobacterial isolates were obtained from different patients at two health care institutions in Lima, Peru. KP1137, KP1139, and EC1141 were isolated from urine; KP1138 and CP1140 were isolated from blood and bronchial secretions, respectively. Identification and susceptibility testing were performed on the Vitek2 compact system (bioMérieux, France). The results were analyzed using criteria from the Clinical and Laboratory Standards Institute (CLSI) (14). Phenotypic detection of carbapenemases was performed using the Triton-Hodge test (15). Detection of OXA-48-like carbapenemase production was performed using the RESIST-4 OKNV (OXA-48-like, KPC, NDM, VIM) immunochromatographic lateral flow assay (Coris BioConcept, Belgium) following the manufacturer’s instructions. Molecular confirmation of OXA-48-like genes was performed by conventional PCR using the primers 5′-ATGCGTGTATTAGCCTTATCGG-3′ (forward) and 5′-TGAGCACTTCTTTTGTGATG-3′ (reverse), yielding a 775-bp amplicon (16).

DNA was extracted from overnight cultures in LB broth using the GeneJET DNA purification kit (Thermo Fisher Scientific, USA), according to the manufacturer’s instructions. The DNA concentration was measured using a Qubit 4 fluorometer (Life Technologies, USA). Genomic libraries were prepared using the Nextera XT DNA library preparation kit (Illumina, USA) and sequenced on an Illumina MiSeq instrument, generating 2 × 250-nucleotide (nt) reads. The read quality was assessed using FastQC v0.11.5 (https://www.bioinformatics.babraham.ac.uk/projects/fastqc/). Adapters and low-quality reads were removed using Trimmomatic v0.39 (17), and *de novo* assembly was performed using SPAdes v3.15.2 (18). The assembled contigs were polished by paired read mapping using Pilon v1.24 (19), and genome assembly metrics were generated using QUAST v5.0.2 (https://github.com/ablab/quast). Genomic multilocus sequence type (MLST), virulence, and plasmid profiles were obtained using MLST v2.22.0 (https://github.com/tseemann/mlst) and the associated PlasmidFinder and VFDB databases (http://www.mgc.ac.cn/VFs/). Contigs were screened for antimicrobial resistance genes (ARGs) using ABRicate v1.0.1 (https://github.com/tseemann/abricate) by querying the NCBI and ResFinder databases. Klebsiella locus profile species were determined using Kleborate v0.2.0 (20). Serotype profiling for Escherichia coli isolates was defined using SerotypeFinder v2.0 and SeqSero v1.0. *In silico* typing of E. coli was performed using ClermonTyping v1.3.0 (21). The genetic context of *bla*_OXA-181_ was determined by extracting the contigs that carried this gene and annotating them using Prokka v1.14.6 (22) with a BLASTP/BLASTN combination. Transposable genetic elements (Tn) were investigated using Integrall (http://integrall.bio.ua.pt/), Mobile Element Finder v1.0.1 (https://cge.food.dtu.dk/services/MobileElementFinder/), and ISfinder (https://isfinder.biotoul.fr/) and were finally identified in the Transposon Registry (https://transposon.lstmed.ac.uk/tn-registry) (23). The Prokka GBK annotation files were parsed for Easyfig image output (https://mjsull.github.io/Easyfig/).

The isolates were identified as Klebsiella pneumoniae (*n* = 3), Citrobacter portucalensis (*n* = 1), and Escherichia coli (*n* = 1). K. pneumoniae isolates KP1137 and KP1138 were resistant to ertapenem and susceptible to second-, third-, and fourth-generation cephalosporins. KP1139 was resistant to all β-lactams, including ertapenem, imipenem, and meropenem. It was also resistant to fluoroquinolones, tetracyclines, and sulfamethoxazole/trimethoprim, only showing susceptibility to aminoglycosides. The isolates E. coli EC1141 and *C. portucalensis* CP1140 were resistant to ertapenem (see Table S1 in the supplemental material). All isolates were identified as carbapenemase producers using the Triton-Hodge test. Moreover, all were positive for the RESIST-4 OKNV immunochromatography test and *bla*_OXA-48_-like genes using conventional PCR. All isolates had multidrug-resistant (MDR) phenotypes. MDR *bla*_OXA_ producers are a rising health care issue that lengthens hospital stays and comorbidities because of their resistance to the antibiotics used to treat CPE infections (24).

**FIG 1:**
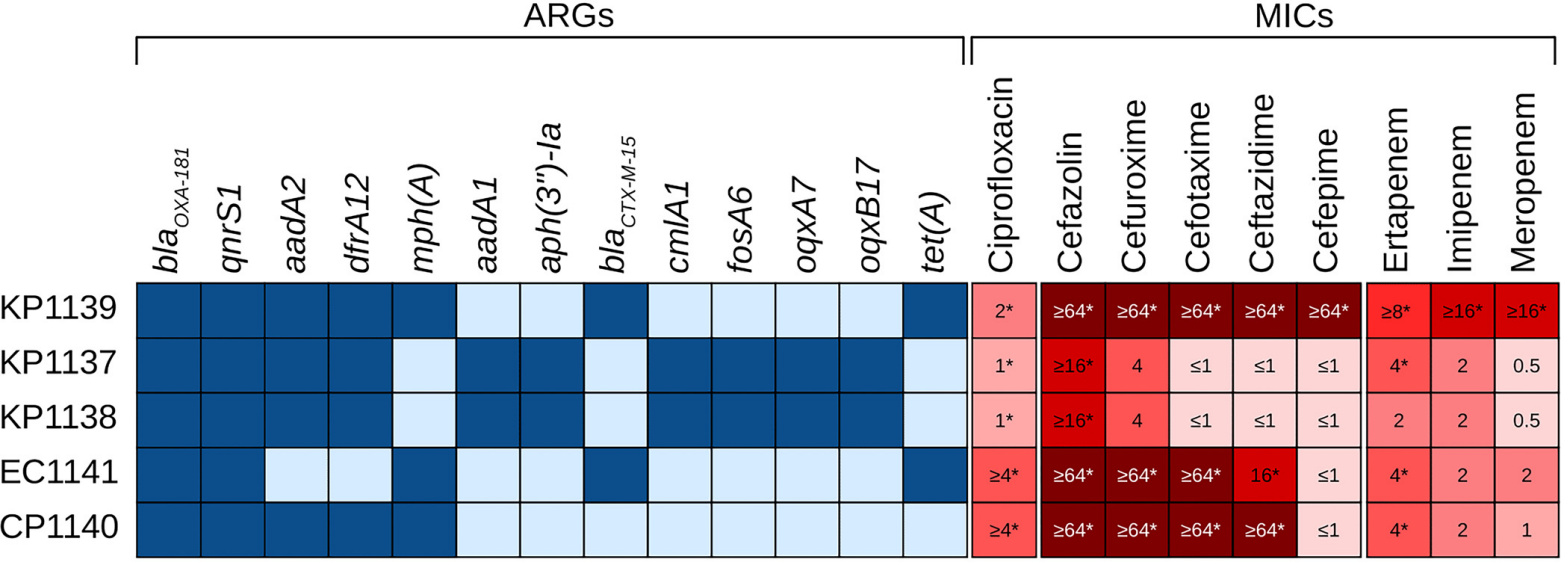
Antimicrobial resistance genes and MIC values of our *Enterobacteriaceae* isolates. ARG, antimicrobial resistance gene; KP, Klebsiella pneumoniae; EC, Escherichia coli; CP, Citrobacter portucalensis. The asterisks (*) indicate the MIC values for each antibiotic.

Whole-genome sequencing revealed that E. coli isolate EC1141 belongs to sequence type 131 (ST131), serotype O50:H4, and Clermont phylogroup B2. K. pneumoniae isolates KP1137 and KP1138 were identified as ST25, with capsular type wzi72, capsular locus KL2, and antigen locus O1. K. pneumoniae KP1139 was identified as ST1174, with capsular type wzi275, KL38, and O12. The *C. portucalensis* isolate was identified as ST129. Thirty-one ARG hits were identified across all isolates, with a mean of 11.8 ARGs per genome. The *bla*_OXA-181_ gene was identified in all genomes, being the only carbapenemase gene in the data set. Additionally, K. pneumoniae KP1139 and E. coli EC1141 exhibited the extended spectrum β-lactamase (ESBL) gene *bla*_CTX-M15_ (Fig. 1). ARGs associated with resistance to aminoglycosides, quinolones, amphenicols, fosfomycins, macrolides, tetracyclines, sulfonamides, and trimethoprim were also identified (Table S2).

**FIG 2:**
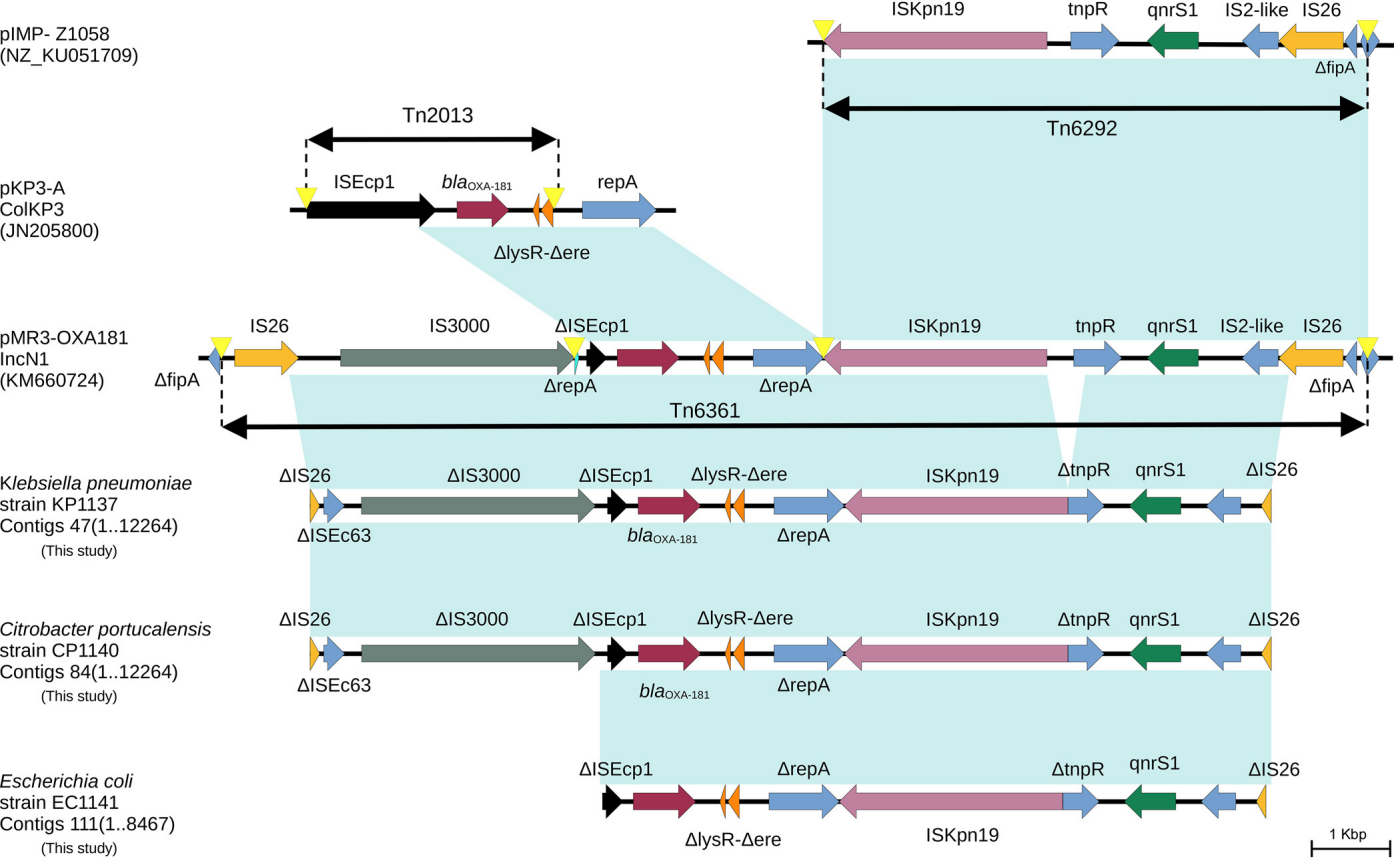
Genetic context of *bla*_OXA-181_-carrying Tn*6361* (GenBank accession number KM660724) transposon structures in our *Enterobacterales* isolates. Transposons Tn*2013* (JN205800) and Tn*6292* (NZ_KU051709) were included for comparison. The same genetic environment of the *bla*_OXA-181_-carrying Tn*6361* in K. pneumoniae KP1137 was found in strains KP1138 and KP1139. Shading indicates 100% sequence similarity.

The *bla*_OXA-181_ gene has been reported in K. pneumoniae (13), E. coli (25), and *Citrobacter* sp. (26) isolates. The plasmid incompatibility group IncX3 was identified in all our isolates, in line with previous studies in CPE (27). This plasmid group is involved in the worldwide dissemination of *bla*_OXA-181_ (28), and its presence in these CPE isolates suggests extended dissemination of *bla*_OXA-181_ in Peru. The *bla*_OXA-181_ gene was located in a truncated transposon Tn*6361*, flanked by two ΔIS*26* insertion sequences. The *qnrS1* gene was detected downstream of *bla*_OXA-181_, conferring fluoroquinolone resistance in all isolates. The *bla*_OXA-181_ gene was flanked upstream by the Tn*3*-like ΔIS*3000* truncated insertion sequence, followed by the truncated IS*Ecp1* gene, and downstream by the Δ*lysR* (transcriptional regulator)-Δ*ere* (erythromycin esterase)-Δ*repA* (Col replicase) gene cluster (Fig. 2). This fragment is followed by the IS*Kpn19*-*tnpR*-*qnrS1*-IS*2*-like gene set, whose structure is similar to Tn*6292*. This region was also compared to pRIVM_C011701_1 (GenBank accession number CP068340; 100% coverage and 100% sequence similarity) and pKP709-OXA181 (MN227183; 100% coverage and 100% sequence similarity).

In summary, we present the first report of MDR OXA-181-producing *Enterobacterales* isolates from Peru and highlight the use of WGS to monitor the dissemination of CPE isolates. Our results suggest the emergence and wide distribution of *bla*_OXA-181_ in association with the *qnrS1* gene on IncX3-type plasmids, which could represent the primary vector for the spread of *bla*_OXA-181_ in Latin America.

### Ethical approval.

The study protocol was approved by the Institutional Ethics Committee of the Universidad Peruana Cayetano Heredia (SIDISI 207858). Patient information was anonymized and deidentified before analysis.

### Data availability.

The raw read files and assemblies for the five isolates are available at NCBI under BioProject accession number PRJNA860216.
